# Brain microstructural alterations and cognitive impairment in obstructive sleep apnea: a diffusion kurtosis imaging study

**DOI:** 10.3389/fneur.2026.1750749

**Published:** 2026-02-16

**Authors:** Ning Zhang, Shiyu He, Jinxia Guo, Ailian Xiao, Juntong Li, Kun Peng

**Affiliations:** 1 Department of Radiology, The Sixth Hospital of Shanxi Medical University (General Hospital of TISCO), Taiyuan, China; 2 College of Medical Imaging, Shanxi Medical University, Taiyuan, China; 3 GE Healthcare, Beijing, China; 4 Department of Respiratory and Critical Care Medicine, The Sixth Hospital of Shanxi Medical University (General Hospital of TISCO), Taiyuan, China

**Keywords:** axial kurtosis, cognitive function, diffusion kurtosis imaging, obstructive sleep apnea, radial kurtosis

## Abstract

**Background:**

This study aimed to investigate brain microstructural alterations and their association with neurocognitive impairment in adults with moderate to severe obstructive sleep apnea (OSA) using diffusion kurtosis imaging (DKI), to better understand the neuropathological mechanisms contributing to cognitive decline in OSA.

**Materials and methods:**

40 OSA patients and 40 matched healthy controls (HCs) underwent cognitive assessments using the Montreal Cognitive Assessment (MoCA) and MRI scans including DKI and 3D T1-weighted imaging. DKI were processed to generate kurtosis parameter maps, including axial kurtosis (AK) and radial kurtosis (RK). Brain region values were extracted using SPM12. Group comparisons were conducted for cognitive scores and kurtosis values. Receiver operating characteristic (ROC) curves were used to assess the diagnostic performance of imaging biomarkers. Partial correlation analysis examined relationships between imaging metrics, cognitive scores, and sleep-related variables. Multiple comparisons were corrected using the false discovery rate (FDR) method.

**Results:**

The OSA group showed increased AK in 9 brain regions and decreased RK in 28 regions. MoCA scores, particularly in visual space and executive function, abstraction, and delayed recall, were significantly lower in the OSA group. ROC analysis showed that RK in specific brain regions had strong diagnostic accuracy for OSA (AUC = 0.817). Lower oxygen saturation (LSpO_2_) was associated with altered kurtosis values in key regions related to cognition. Cognitive scores were positively correlated with RK values in regions such as the frontal cortex, cingulate cortex, and hippocampus.

**Conclusion:**

DKI effectively detects microstructural brain changes in OSA patients. These alterations are associated with cognitive decline, providing valuable insights into the potential mechanisms underlying neurocognitive impairments in OSA.

## 1 Introduction

Obstructive sleep apnea is a sleep disorder characterized by loud snoring, recurrent episodes of apnea or hypopnea, and frequent nocturnal arousals (1). It is estimated that approximately 1 billion adults worldwide are affected by OSA, with its prevalence steadily increasing, thereby making it a significant health issue (2). Growing evidence suggests that chronic sleep fragmentation, recurrent intermittent hypoxia, and hypercapnia associated with OSA can lead to secondary damage to the central nervous system. This damage results in alterations in brain microstructure and subsequent cognitive impairment, which substantially impact patients’ quality of life and occupational performance (3, 4).

From a pathophysiological perspective, repeated cycles of hypoxia-reoxygenation in OSA can induce oxidative stress and vascular endothelial dysfunction, leading to impaired cerebral autoregulation and chronic hypoperfusion. These processes may activate neuro-inflammatory responses, including microglial activation and cytokine release, which in turn can disrupt oligodendrocyte function and myelin homeostasis, thereby promoting demyelination. Simultaneously, hypoxia-related metabolic disturbances and inflammatory signaling can impose sustained stress on axonal structures, resulting in axonal microstructural damage. White matter is particularly vulnerable to hypoxic injury, and the disruption of myelin integrity and axonal organization may compromise neural signal transmission and large-scale brain network connectivity, providing a structural basis for OSA-related cognitive dysfunction (5).

In recent years, diffusion tensor imaging (DTI) has been widely used to investigate brain alterations in patients with OSA, primarily revealing reduced white matter fiber integrity (6, 7). However, DTI is based on a Gaussian diffusion model, which assumes unrestricted water diffusion and is inherently limited in its ability to characterize complex tissue microenvironment, which is influenced by factors such as cellular heterogeneity, myelin architecture, and axonal organization. Consequently, DTI may not fully capture subtle or complex microstructural abnormalities associated with OSA. DKI, an extension of DTI, models non-Gaussian water diffusion behavior and provides enhanced sensitivity to the complexity of tissue microstructure. Prior studies have demonstrated that DKI is more sensitive than DTI in detecting microstructural abnormalities in various neurological conditions (8). In particular, AK has been linked to axonal integrity and intra-axonal micro-environmental changes, while RK is thought to be more sensitive to myelin-related and glial alterations, making DKI particularly well-suited for probing OSA-related white matter injury (9).

Therefore, the present study employed DKI to quantitatively assess brain tissue microstructural alterations in patients with moderate-to-severe OSA and to investigate the relationship between DKI -derived metrics, and cognitive performance. This study aims to elucidate the neuropathological mechanisms underlying cognitive impairment in OSA patients and to provide an imaging-based framework for a comprehensive clinical evaluation.

## 2 Materials and methods

### 2.1 Participants

A total of 40 patients with newly diagnosed, untreated moderate-to-severe OSA were enrolled, along with 40 HCs matched for age, sex, and years of education.

The following inclusion criteria were applied for participant selection in both the OSA and HC groups. For the OSA group, participants were required to meet the following criteria: (1) a diagnosis of OSA confirmed by overnight Polysomnography (PSG), with an Apnea-Hypopnea Index (AHI) of ≥15 events per hour; (2) age between 20 and 65 years; (3) right-handed; and (4) body mass <125 kg. For the HC group, although PSG was not performed, participants underwent structured screening procedures, including detailed clinical interviews to assess sleep-related symptoms. In addition, family members were interviewed to determine the presence of chronic snoring or witnessed apneas. Participants in the HC group were required to meet the following criteria: (1) no sleep disturbances, as confirmed by family members, with no reports of snoring, apnea, daytime sleepiness, or other symptoms; (2) age between 20 and 65 years; (3) right-handed; and (4) body mass <125 kg.

Participants were excluded based on the following criteria: (1) Presence of other sleep and breathing disorders beyond OSA, such as primary insomnia; (2) History of nervous system diseases, including massive cerebral infarction, brain tumors, or brain injury; (3) Severe anxiety, depression, or other mental health disorders; (4) Chronic conditions such as severe hypertension or diabetes; (5) Claustrophobia or other contraindications that would prevent participation in MRI examinations.

The research plan received ethical approval from the Medical Ethics Committee of the General Hospital of TISCO (K202210). Informed consent was obtained from all participants.

### 2.2 PSG assessment

Overnight PSG examinations were conducted in the sleep monitoring room of the department of respiratory medicine. The Compumedics Grael PSG system was adopted for 8 h continuous overnight monitoring. The hardware configuration included the Grael digital acquisition host, electroencephalogram (EEG) electrodes positioned according to the 10–20 international system, bilateral electrooculogram (EOG) electrodes, electrocardiogram (ECG) electrodes, submental electromyogram (EMG) electrodes, thoracic and abdominal effort transducers, oronasal airflow sensors, fingertip pulse oximetry probes, bilateral leg movement sensors, and body position sensors. The integrated Profusion PSG analysis software was utilized for real-time waveform monitoring, automated sleep staging, and event annotation.

From the PSG datasets, the following parameters were extracted: AHI (events/h), lowest oxygen saturation (lowest SpO_2_, %), mean oxygen saturation (mean SpO_2_, %), oxygen desaturation index (ODI, events/h), sleep efficiency (SE, %), total sleep time (TST, min).

### 2.3 Assessment of cognitive function

On the day of the MRI examination, participants underwent cognitive assessment using the MoCA in a quiet room. This evaluation assessed various cognitive domains, including visual–spatial and executive functions, attention, language and abstraction, and memory. The MoCA total score ranges to 30 points, with 26 or below indicating potential cognitive dysfunction. The assessment was conducted by a professionally trained neurologist who was blinded to the participants’ clinical information.

### 2.4 MRI data acquisition

All subjects underwent head MRI using a GE 3.0 T SIGNA Pioneer MR scanner with a 21-channel head coil. The examination included conventional MRI scanning to check for brain parenchymal lesions, followed by transverse DKI and 3D T1-weighted imaging (3D T1WI). For the 3D T1WI scan, the 3D T1 MP-RAGE sequence was used with parameters: TR/TE = 2,384/2.5 ms, TI = 1,000 ms, flip angle = 8°, field of view = 240 × 240 mm, matrix = 224 × 224, slice thickness = 1.1 mm, with a total of 146 slices. The scanning time for 3D T1WI was 4 min and 56 s. The DKI scan was performed with parameters: TR/TE = 12,000/88.7 ms, field of view = 240 × 240 mm, matrix = 120 × 120, slice thickness = 5.0 mm, slice spacing = 1.5 mm, *B*-values of 0 s/mm^2^, 1,000 s/mm^2^, and 2,000 s/mm^2^, with 25 diffusion encoding directions for each non-zero *B*-value. The total scanning time for DKI was 10 min and 48 s.

### 2.5 Data processing

**Figure 1:**
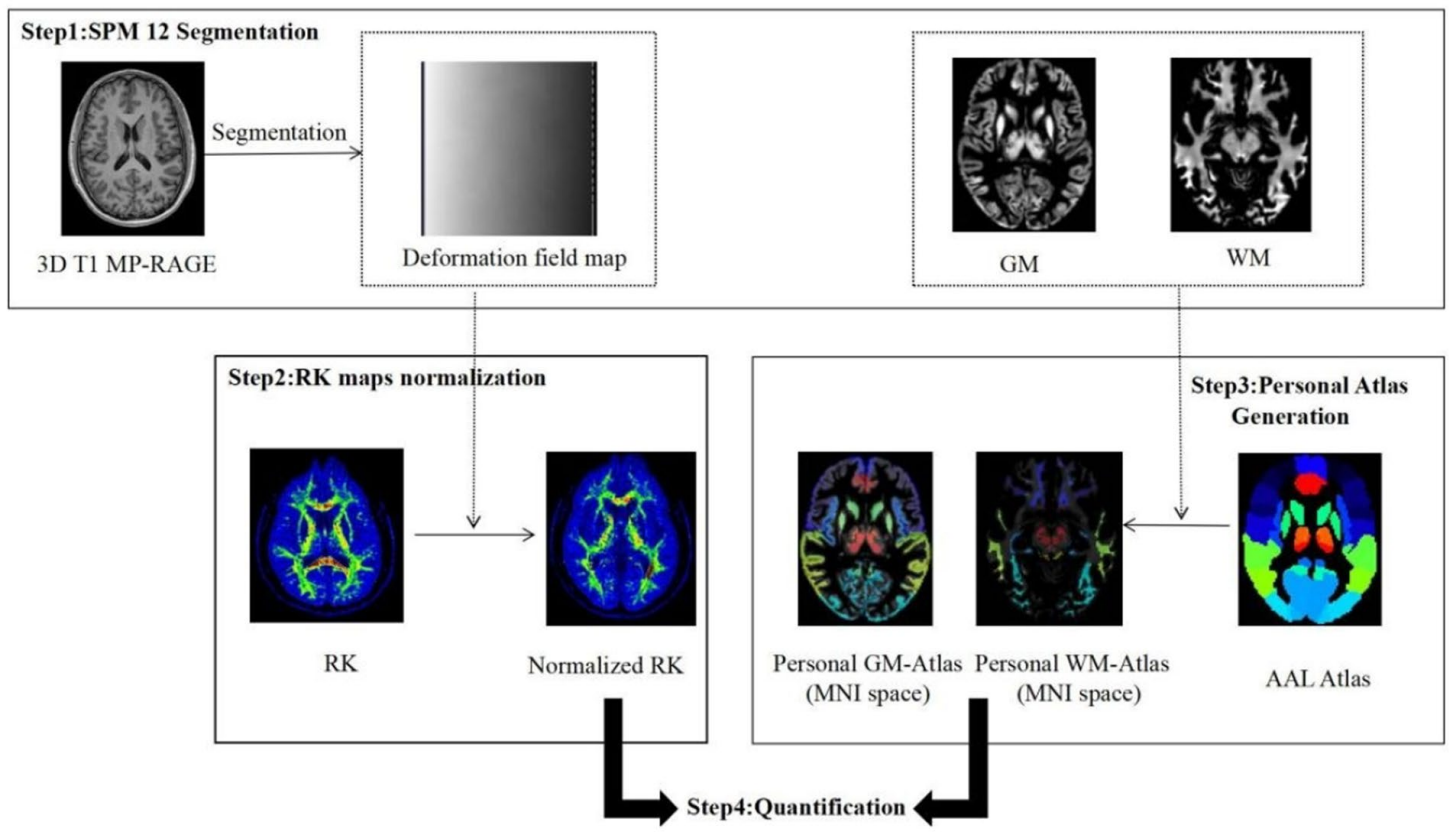
DKI kurtosis parameter post-processing flow chart (taking RK parameter chart as an example). AAL, Automated Anatomical Labeling Atlas; GM, Gray matter; MNI, Montreal Neurological Institute; RK, Radial kurtosis; WM, White matter.

The DKI images were processed using iQuant software (GE Healthcare, China) to generate diffusion kurtosis parameter maps, including AK and RK. These maps were then analyzed with SPM12 based on the Automatic Anatomical Labeling Atlas (AAL3). The procedure involved segmenting the 3D T1-weighted images into gray and white matter and then generating a deformation mapping field from the original scanning data to the standard Montreal Neurological Institute (MNI) space. The AK and RK maps were registered to the 3D T1-weighted images and transformed into MNI space. Next, the segmented gray matter/white matter map and the standardized AK and RK maps were resampled to match the resolution of the AAL map (1 mm × 1 mm × 1 mm). Brain regions were identified based on the overlap of the brain atlas with gray or white matter, and corresponding AK and RK values were extracted. To reduce recognition errors, small brain regions were combined and quantified. The general flow of the data processing is illustrated in Figure 1.

### 2.6 Statistical analysis

Statistical analyses were performed using SPSS 26.0. The Shapiro-Wilk test was used to assess the normality of continuous variables for each region of interest. Data following a normal distribution are presented as mean ± standard deviation (*x̄* ± *s*), and between-group comparisons were conducted using independent-samples *t* tests. Data that did not conform to a normal distribution are presented as median (*P*_25_, *P*_75_) and were analyzed using the Mann-Whitney *U* test. Categorical data, such as sex, are expressed as proportions and were compared between groups conducted using the Chi-square (*χ*^2^) test. To control for multiple comparisons across brain regions, the Benjamini-Hochberg FDR correction was applied, with a *q*-value threshold of 0.05. ROC curve analyses were performed to evaluate the diagnostic performance of DKI parameters. Specifically, kurtosis values from brain regions showing significant group differences were used as test variables, with group status (OSA vs. HCs) as the state variable. These ROC analyses were conducted for exploratory purposes to assess the discriminatory potential of selected DKI parameters. Diagnostic performance was quantified by calculating sensitivity, specificity, and the area under the ROC curve (AUC). Partial correlation analyses were conducted within the OSA group to examine the relationships between DKI kurtosis parameters and sleep-related indices, including the AHI and lowest oxygen saturation (LSpO_2_), as well as cognitive performance measured by the MoCA score. These analyses were adjusted for potential confounding factors, including age, Body Mass Index (BMI), and years of education. Multiple comparisons in correlation analyses were also corrected using the FDR method, with statistical significance set at *p* < 0.05.

The primary outcomes of this study included the difference in DKI kurtosis parameters (AK and RK) between OSA patients and HCs, and correlations between DKI parameters and sleep-related variables, cognitive performance (MoCA scores). Secondary outcome was the exploratory ROC analyses evaluating the discriminatory ability of selected DKI parameters.

## 3 Results

### 3.1 Demographic and clinical characteristics of OSA patients and controls

Table 1 summarizes the demographic characteristics, and clinical variables, and MoCA scale scores of the OSA and HC groups. No significant differences were observed in age, sex, or years of education (all *p* > 0.05). In contrast, BMI was significantly higher in the OSA group compared with the HC group (*p* < 0.001).

**Table 1:** Comparison of demographic characteristics, clinical data and MoCA scale scores between the two groups.

Characteristics		OSA (*n* = 40)	HC (*n* = 40)	Statistical value	*p*
Demographic	Age (year)	46.08 ± 10.11	43.78 ± 9.04	−1.072^a^	0.287
	Sex (male : female)	35 : 5	28 : 12	3.660^b^	0.056
	BMI (kg/m^2^)	28.15 ± 2.99	25.03 ± 3.53	−4.254^a^	<0.001^*^
	Education (year)	14.00 (11.00, 15.00)	14.00 (11.00, 15.00)	−0.891^c^	0.373
Overnight PSG data	AHI (events/h)	38.40 ± 17.21	—	—	—
	LSpO_2_ (%)	71.48 ± 12.05	—	—	—
	Mean SpO_2_ (%)	89.73 ± 5.89	—	—	—
	ODI (events/h)	34.81 ± 19.17	—	—	—
	SE (%)	73.45 ± 22.29	—	—	—
	TST (min)	526.46 ± 128.57	—	—	—
MoCA assessment data	Total MoCA scores	22.88 ± 2.49	26.83 ± 1.65	8.361^a^	<0.001^*^
	Visuospatial	3.00 (3.00, 4.00)	4.00 (4.00, 5.00)	−2.800^b^	<0.001^*^
	Naming	3.00 (3.00, 3.00)	3.00 (3.00, 3.00)	−0.356^b^	0.722
	Attention	5.00 (5.00, 6.00)	6.00 (5.00, 6.00)	−0.923^b^	0.356
	Language	2.00 (1.25, 3.00)	2.00 (2.00, 2.00)	−0.827^b^	0.408
	Abstraction	1.00 (0.25, 2.00)	2.00 (2.00, 2.00)	−4.736^b^	<0.001^*^
	Delayed recall	2.00 (1.00, 3.00)	4.00 (4.00, 4.00)	−6.166^b^	<0.001^*^
	Orientation	6.00 (6.00, 6.00)	6.00 (6.00, 6.00)	−1.388^b^	0.165

a Student’s *t* test, the statistical value is the *t* value.

b Chi-squared test, the statistical value is *χ*^2^ value.

c Mann-Whitney *U* test, the statistical value is the *z* value.

AHI, Apnea-Hypopnea Index; BMI, Body Mass Index; HC, Healthy control; LSpO_2_, Lowest oxygen saturation; MoCA, Montreal Cognitive Assessment; OSA, Obstructive sleep apnea; ODI, Oxygen desaturation index; PSG, Polysomnography; SE, Sleep efficiency; TST, Total sleep time.

The total MoCA score was significantly lower in the OSA group than in the HC group, indicating poorer global cognitive performance at the screening level. In addition, scores in the visual space and executive function, delayed recall, and abstraction were significantly reduced in OSA patients (all *p* < 0.05).

### 3.2 Differential AK and RK values in brain regions between OSA patients and controls

**Figure 2:**
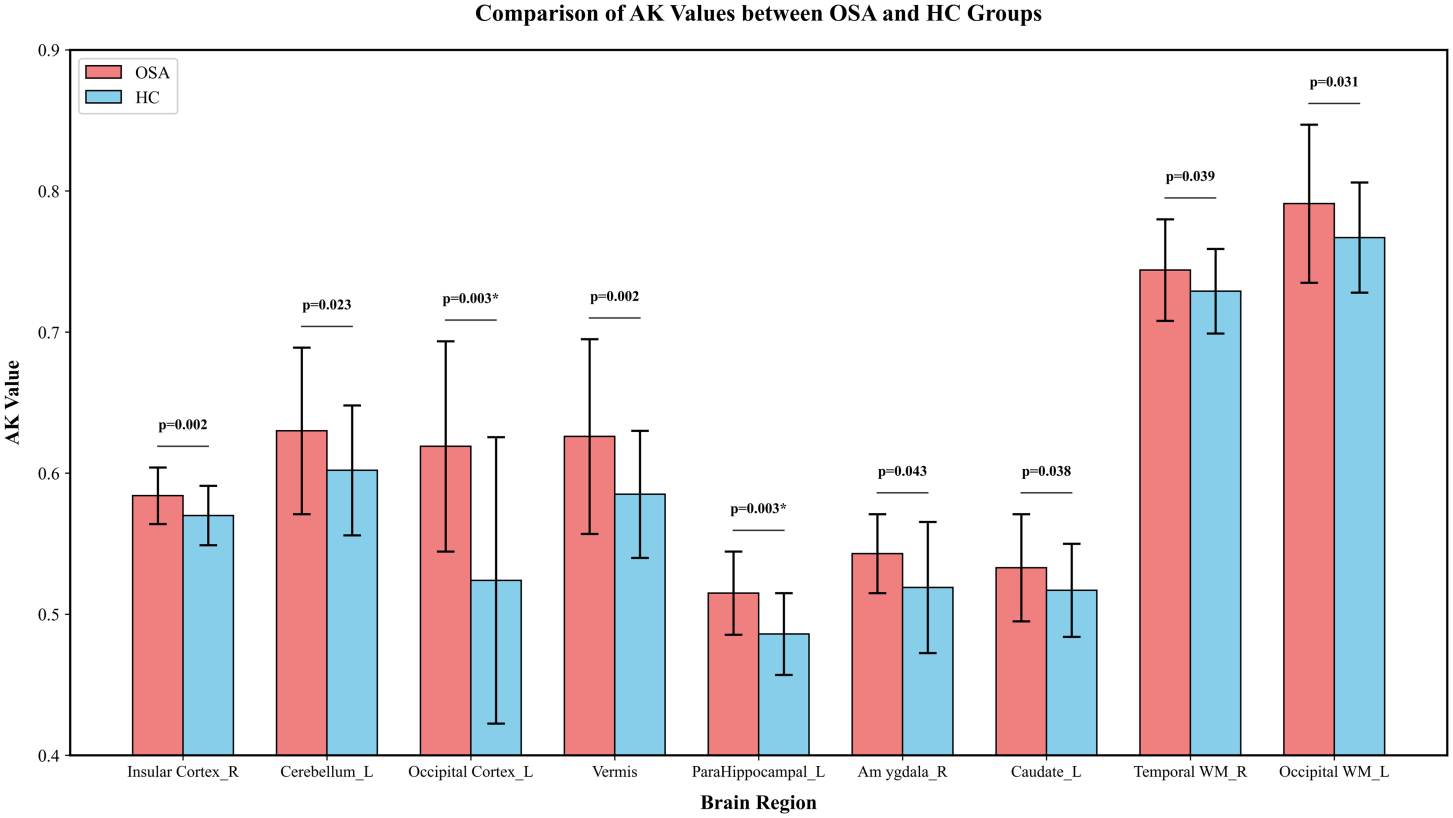
Comparison of AK values between OSA and HC groups across brain regions. Only brain region with significant difference in AK value was plotted. Bars represent the central values (mean or median), and error bars indicate variability, shown as standard deviation for mean-based data or half of the interquartile range for median-based data. *p*-values indicate between-group comparisons, *p* < 0.05. ^*^Significant after correction for FDR. AK, Axial kurtosis; HC, Healthy control; L, Left; OSA, Obstructive sleep apnea; R, Right; WM, White matter.

**Figure 3:**
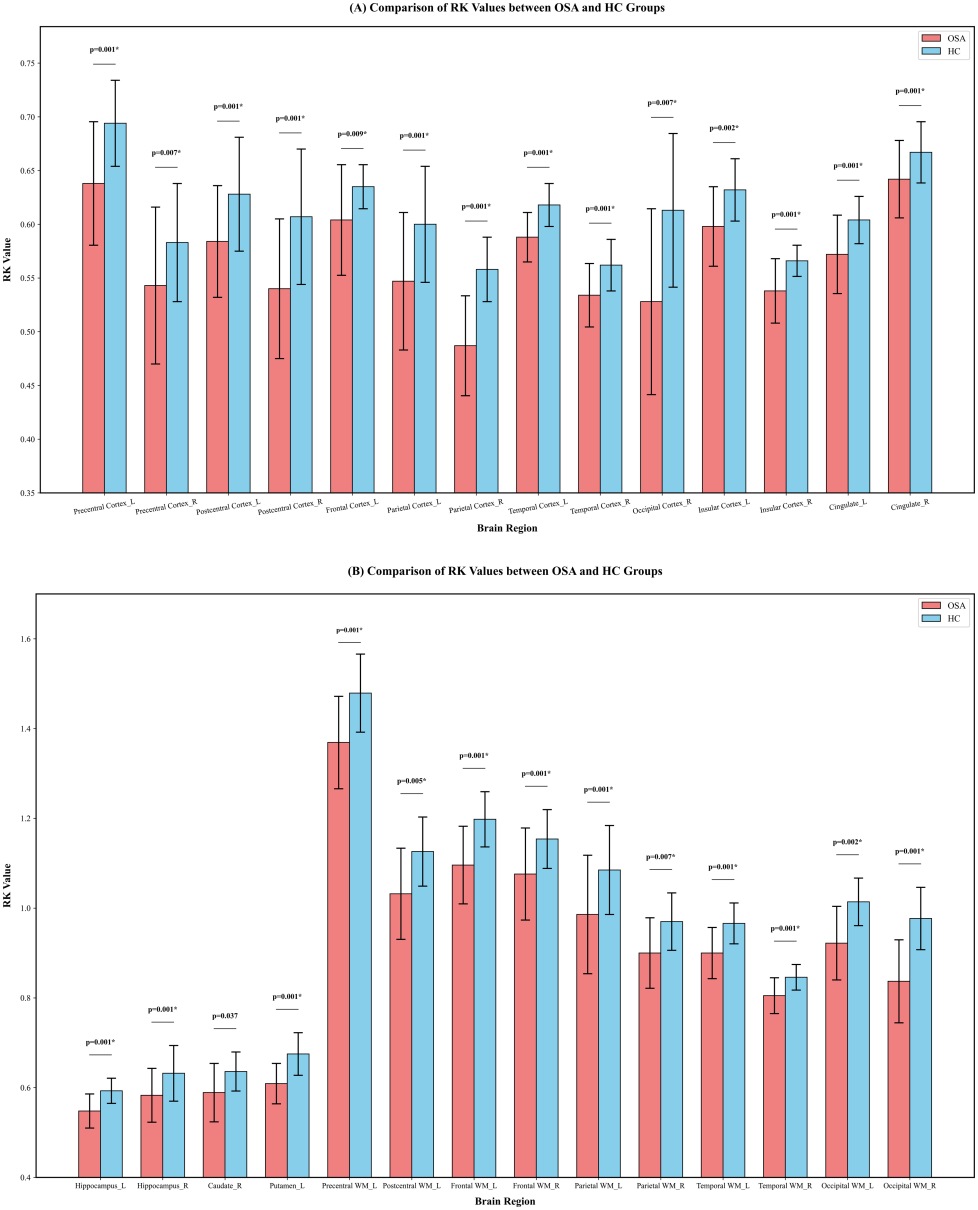
Comparison of RK values between OSA and HC groups across brain regions. **(A)** RK values in cortical regions. **(B)** RK values in white matter regions. Bars represent the central values (mean or median), and error bars indicate variability, shown as standard deviation for mean-based data or half of the interquartile range for median-based data. *p*-values indicate between-group comparisons, *p* 0.05. ^*^Significant after correction for FDR. HC, Healthy control; L, Left; OSA, Obstructive sleep apnea; RK, Radial kurtosis; R, Right; WM, White matter.

Compared to the HC group, the AK values were significantly increased in several brain regions of the OSA group (*p* < 0.05). These findings suggest that AK alterations in OSA patients were relatively region-specific, primarily involving limbic structures, cerebellar regions, and occipital cortices. Details are provided in Figure 2. In contrast, the RK values were significantly decreased in several brain regions of the OSA group (*p* < 0.05). Unlike AK, RK alterations in OSA patients were more extensive and widely distributed, involving multiple cortical regions and widespread white matter tracts. Details are provided in Figure 3.

### 3.3 Correlation of DKI kurtosis parameters and sleep breathing parameters in OSA patients

After adjusting for potential confounders such as age, BMI, and years of education, LSpO_2_ in the OSA group was negatively correlated with the AK value of the left para-hippocampal gyrus (*r* = −0.384, *p* < 0.05) and positively correlated with the RK value of bilateral parietal cortex, right insular cortex and occipital white matter (*r* = 0.362, 0.402, 0.394, 0.338, *p* < 0.05). Details are provided in Table 2.

**Table 2:** Correlation of DKI kurtosis parameters and sleep breathing indices as well as MoCA score in OSA patients.

Brain region		AHI		LSpO_2_		Brain region	MoCA		Visuospatial		Delayed recall	
		*r*	*p*	*r*	*p*		*r*	*p*	*r*	*p*	*r*	*p*
AK	ParaHippocampal_L	0.158	0.350	−0.384	0.019^*^							
RK	Parietal cortex_L	−0.290	0.082	0.362	0.028^*^	Frontal cortex_L	0.373	0.023^*^	0.188	0.264	0.343	0.037^*^
	Parietal cortex_R	−0.221	0.189	0.402	0.014^*^	Cingulate_R	0.248	0.139	0.345	0.037^*^	0.244	0.146
	Insular cortex_R	−0.272	0.103	0.394	0.016^*^	Hippocampus_R	0.263	0.116	0.226	0.179	0.341	0.039^*^
	Occipital WM_R	−0.175	0.301	0.338	0.041^*^	Frontal WM_L	0.205	0.223	0.055	0.746	0.348	0.035^*^
						Temporal WM_L	0.311	0.061	0.006	0.972	0.374	0.023^*^

^*^At the level of 0.05 (double tail), the correlation is significant. AHI, Apnea-Hypopnea Index; AK, Axial kurtosis; L, Left; LSpO_2_, Lowest oxygen saturation; MoCA, Montreal Cognitive Assessment; OSA, Obstructive sleep apnea; R, Right; RK, Radial kurtosis; WM, White matter.

### 3.4 Correlation of DKI kurtosis parameters and MoCA score in OSA patients

After adjusting for age, BMI, and years of education, the MoCA total score in the OSA group was positively correlated with the RK value of the left frontal cortex (*r* = 0.373, *p* < 0.05). Additionally, visual space and executive function were positively correlated with the RK value of the right cingulate gyrus (*r* = 0.345, *p* < 0.05). Delayed recall showed associations with the RK values of the left frontal cortex, right hippocampus, and left front-temporal white matter (*r* = 0.343, 0.341, 0.348, 0.374, *p* < 0.05). These correlations were primarily observed in frontal, hippocampal, and cingulate regions, which are closely related to cognitive control and memory processing. Details are provided in Table 2.

### 3.5 ROC curve analysis of DKI-related parameters in the diagnosis of OSA

**Figure 4:**
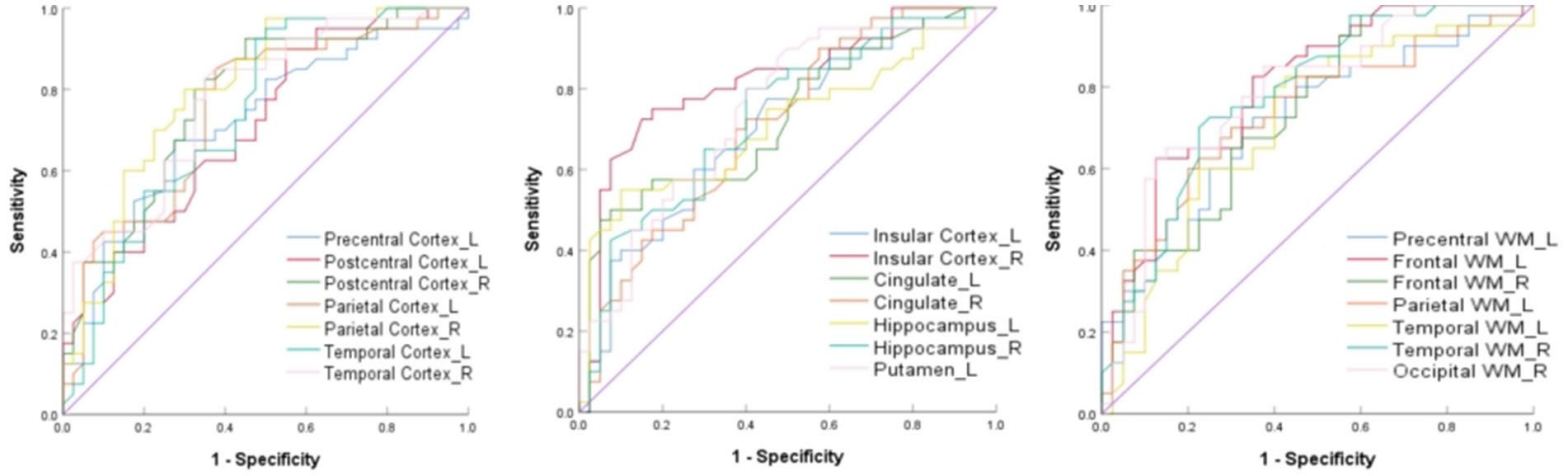
ROC curves of brain regions with different RK values after FDR correction to distinguish OSA from HCs. FDR, False discovery rate; RK, Radial kurtosis; ROC, Receiver operating characteristic; OSA, Obstructive sleep apnea.

Among the regions showing significant RK alterations, several demonstrated moderate discriminatory ability for OSA in exploratory ROC analyses, with the right insular cortex demonstrating the highest AUC. Detailed ROC results are shown in Table 3 and Figure 4.

**Table 3:** Diagnostic efficiency of RK parameters for OSA.

Brain region	AUC (95% CI)	Sensitivity	Specificity
Precentral cortex_L	0.720 (0.607–0.833)	0.675	0.725
Postcentral cortex_L	0.708 (0.596–0.820)	0.900	0.450
Postcentral cortex_R	0.777 (0.675–0.879)	0.925	0.550
Parietal cortex_L	0.751 (0.643–0.859)	0.850	0.625
Parietal cortex_R	0.799 (0.702–0.897)	0.800	0.700
Temporal cortex_L	0.739 (0.630–0.849)	0.925	0.525
Temporal cortex_R	0.778 (0.678–0.878)	0.825	0.650
Insular cortex_L	0.705 (0.592–0.819)	0.775	0.550
Insular cortex_R	0.817 (0.720–0.914)	0.725	0.850
Cingulate_L	0.726 (0.615–0.838)	0.500	0.925
Cingulate_R	0.708 (0.595–0.821)	0.900	0.425
Hippocampus_L	0.713 (0.598–0.828)	0.550	0.900
Hippocampus_R	0.730 (0.620–0.841)	0.800	0.600
Putamen_L	0.743 (0.635–0.852)	0.800	0.600
Precentral WM_L	0.716 (0.603–0.828)	0.700	0.675
Frontal WM_L	0.799 (0.703–0.896)	0.625	0.875
Frontal WM_R	0.734 (0.625–0.843)	0.925	0.450
Parietal WM_L	0.735 (0.624–0.845)	0.600	0.800
Temporal WM_L	0.708 (0.591–0.824)	0.800	0.600
Temporal WM_R	0.779 (0.678–0.880)	0.700	0.775
Occipital WM_R	0.784 (0.682–0.886)	0.625	0.875

L, Left; OSA, Obstructive sleep apnea; R, Right; WM, White matter.

## 4 Discussion

This study utilized DKI and the whole brain map analysis template from the Montreal Institute of Neurology to examine the brain alterations. The results revealed abnormal changes in DKI parameters (AK, RK) across several brain regions in OSA patients, suggesting potential microstructural damage. Correlation analysis further demonstrated that altered cognitive performance at the screening level was associated with microstructural changes in specific brain regions.

Intermittent hypoxemia and sleep fragmentation are key characteristics of OSA, triggering a series of pathophysiological processes such as oxidative stress, neuroinflammation, cerebral hypoperfusion, vascular endothelial dysfunction, and disruption of the blood-brain barrier (10, 11). These pathological changes contribute to varying degrees of brain structure and function alterations. The resulting microstructural damage is thought to be a primary factor contributing to the reduced tissue heterogeneity observed in OSA patients (12).

AK and RK are two DKI metrics derived from a non-Gaussian diffusion model. AK quantifies diffusion kurtosis along the principal direction of axonal fibers and is considered to reflect axonal integrity, whereas RK measures diffusion kurtosis perpendicular to the axonal direction and is thought to be more sensitive to myelin integrity. Owing to the heterogeneous diffusion environment created by cellular membranes and myelin sheaths, radial diffusion exhibits greater non-Gaussian displacement characteristics; consequently, RK, as a radial metric, may be more sensitive to alterations in water molecule diffusion within complex tissue microenvironments (13, 14). In a preliminary study with a small sample size, Tummala et al. (15) reported that AK and RK values in OSA patients showed more widespread alterations across brain regions compared with conventional DTI metrics, underscoring the increased sensitivity of kurtosis-based imaging to microstructural injury.

The direction of change in kurtosis parameters likely reflects the type of microstructural alterations under pathological conditions. During the acute hypoxia stage, axonal and myelin swelling and increased glial cell proliferation contribute to greater tissue heterogeneity, reflected by increases in both AK and RK values. Conversely, under chronic pathological conditions, axonal damage, demyelination, and neuron loss result in reduced tissue heterogeneity, manifesting as decreases in AK and RK values (16). In this study, OSA patients exhibited increased AK values and decreased RK values. The increase in AK may reflect microstructural alterations that are potentially related to early axonal changes. Decreased RK values may be consistent with alterations in myelin integrity, and are commonly interpreted as indirect markers of potential demyelination. RK changes were more pronounced and widespread than AK changes, surpassing the RD and AD values reported by Chen et al. (17) in their DTI-based study of white matter alterations in OSA patients. These findings suggest that myelin sheath injury in OSA may occur earlier and affect a broader range of brain regions than axonal injury, potentially because myelin is more vulnerable to hypoxia than axons. ROC curve analysis further revealed that changes in RK values in specific brain regions showed good discriminatory power for OSA, with the right insular cortex demonstrating the highest diagnostic value. This suggests that RK values could serve as promising imaging biomarkers for distinguishing OSA patients from HCs. It should be emphasized that DKI-derived AK and RK parameters are indirect imaging surrogate markers of tissue microstructure and do not provide direct histopathological evidence of axonal or myelin injury.

In the present study, after adjusting for age, sex, and other potential confounders, the correlation between AHI/LSpO_2_ and AK/RK values in various brain regions of the OSA group was analyzed. The results revealed that LSpO_2_ was negatively correlated with AK values in the left para-hippocampal gyrus and positively correlated with RK values in the bilateral parietal cortex, right insular cortex, and occipital white matter. AHI, which measures OSA severity, and LSpO_2_, representing the minimum blood oxygen level during episodes of hypoxia, both provide comprehensive assessments of OSA severity. These findings suggest that the progression of OSA may be linked to brain tissue damage induced by ischemia and hypoxia (18).

The results of this study indicate that the total MoCA score in the OSA patient group was significantly lower than in the HC group, with significant differences observed in visuospatial ability, executive function, and delayed memory. Furthermore, abstract function was also found to be moderately impaired in OSA patients. Correlation analyses further revealed significant associations between MoCA subdomain scores and brain regions involved in neuropsychological processing and cognitive control, particularly the hippocampus and cingulate gyrus.

The frontal cortex is closely associated with higher-order cognitive functions, including attention, perception, and working memory (19). In OSA patients, recurrent apnea or hypoventilation events during sleep may induce hypoxia and periodic alterations in cerebral hemodynamics, rendering cortical structures vulnerable to nocturnal ischemia and potentially contributing to impaired cognitive performance (20). The hippocampus plays a critical role in neurogenesis and the integrity of the dentate gyrus and hippocampal circuitry, which are essential for learning and memory processes (21). Moreover, the hippocampus is highly susceptible to hypoxia and oxidative stress. Sleep-related hypoxia in OSA may trigger excessive inflammatory cytokine release, leading to cellular dysfunction. These pathological processes may result in chronic injury or even apoptosis of hippocampal neurons, thereby contributing to neurocognitive impairment (22). The complex architecture of white matter fiber tracts underlies their essential role in cognitive function, including the integration and processing of information related to language and auditory perception (23). Disruption of white matter microstructural integrity due to hypoxia, hypercapnia, or altered cerebral perfusion during sleep in OSA patients may affect parietal-occipital-temporal lobe fiber network, potentially contributing to memory dysfunction. Furthermore, the cingulate gyrus is involved in cognitive and emotional processing, particularly executive function (24). The observed decline in information processing speed and executive dysfunction in OSA patients in this study may be related to microstructural alterations in the cingulate gyrus.

Beyond region-specific findings, the brain areas identified in this study can be integrated into broader functional networks implicated in cognition. The frontal and parietal cortices are key components of the frontoparietal control network, which supports executive function and attentional regulation. The hippocampus is a core node of the default mode network and plays a critical role in memory processing. The cingulate gyrus is involved in both cognitive control and salience detection, serving as an important hub linking multiple large-scale networks. Previous studies on functional networks in patients with OSA have revealed a significant reduction in the connectivity of the default mode network and frontoparietal neural network. Such functional connectivity impairments associated with neuropsychological deficits may underlie the cognitive impairments observed in OSA (25). From a network perspective, the observed DKI alterations may reflect distributed microstructural changes affecting network-level connectivity rather than isolated regional abnormalities.

This study has several limitations. First, the relatively small sample size may reduce the representativeness of the findings and limit the statistical power for group comparisons and correlation analyses. Additionally, the absence of PSG monitoring for the HC group may compromise reliability. Second, cognitive function in this study was assessed using screening tools rather than a comprehensive neuropsychological battery. As such, the findings reflect global cognitive performance at the screening level, rather than a clinical diagnosis of mild cognitive impairment or domain-specific cognitive deficits. Moreover, the study uses an ROI-based approach to explore differences between OSA patients and HCs, restricting the analysis to brain regions in the atlas. Future studies should consider voxel-based comparisons or tract-based spatial statistics analysis to identify better-affected brain regions and assess microstructural damage. Lastly, while this study identifies brain regions potentially affected by OSA, it does not clarify the mechanisms through which OSA contributes to neurocognitive impairment in these regions. The duration of OSA was not included as a variable due to the difficulty in accurately determining the disease’s onset. Future longitudinal studies are needed to address this issue. Additional research could benefit from detailed subgroup analyses based on OSA severity, longitudinal tracking of disease progression, and brain connectivity analysis focused on specific regions.

## 5 Conclusion

In conclusion, this study demonstrates that OSA is associated with widespread brain microstructural alterations that are related to cognitive performance. These findings suggest that DKI may be sensitive to early microstructural changes before overt cognitive impairment becomes clinically evident, highlighting its potential relevance for early detection. In addition, exploratory ROC analyses indicated that selected DKI parameters, particularly RK values in specific brain regions, exhibited moderate discriminatory ability between OSA patients and HCs. These results should be interpreted cautiously and viewed as hypothesis-generating rather than indicative of diagnostic utility. Finally, given the potential reversibility of OSA-related neural changes, DKI may also hold promise for monitoring treatment effects, such as responses to continuous positive airway pressure (CPAP) therapy, in future longitudinal studies.

## Data Availability

The original contributions presented in the study are included in the article/Supplementary material, further inquiries can be directed to the corresponding author.
