# Supplementary material for: Brain microstructural alterations and cognitive impairment in obstructive sleep apnea: a diffusion kurtosis imaging study
Source: Front Neurol. 2026 Feb 16;17:1750749. doi: 10.3389/fneur.2026.1750749 (PMC12950540; doi:10.3389/fneur.2026.1750749)
Supplement: Supplementary file 1 [file Table_1.docx]

**Supplementary Table**

Table S1 ANCOVA results of AK values between OSA and HC group after adjusting for covariates

| Brain Region | F(Group) | P value | Partial η² |
| --- | --- | --- | --- |
| Insular Cortex_R | 8.570 | 0.005 | 0.103 |
| Cerebellum_L | 3.562 | 0.063 | 0.045 |
| Occipital Cortex_L | 2.251 | 0.138 | 0.029 |
| Vermis | 5.103 | 0.027 | 0.064 |
| ParaHippocampal_L | 5.540 | 0.021 | 0.069 |
| Amygdala_R | 4.026 | 0.048 | 0.051 |
| Caudate_L | 0.913 | 0.342 | 0.012 |
| Temporal WM_R | 3.977 | 0.050 | 0.050 |
| Occipital WM_L | 1.333 | 0.252 | 0.017 |

Covariates included age, BMI, and years of education. AK, axial kurtosis; HC, healthy control; L = left; OSA, obstructive sleep apnea; R, right; WM, white matter.

Table S2 ANCOVA results of RK values between OSA and HC group after adjusting for covariates

| Brain Region | F(Group) | P value | Partial η² |
| --- | --- | --- | --- |
| Precentral Cortex_L | 10.559 | 0.002 | 0.123 |
| Precentral Cortex_R | 6.165 | 0.015 | 0.076 |
| Postcentral Cortex_L | 10.936 | 0.001 | 0.127 |
| Postcentral Cortex_R | 11.949 | 0.001 | 0.137 |
| Frontal Cortex_L | 7.264 | 0.009 | 0.088 |
| Parietal Cortex_L | 10.031 | 0.002 | 0.118 |
| Parietal Cortex_R | 16.379 | ＜0.001 | 0.179 |
| Temporal Cortex_L | 11.004 | 0.001 | 0.128 |
| Temporal Cortex_R | 19.513 | ＜0.001 | 0.206 |
| Occipital Cortex_R | 7.758 | 0.007 | 0.094 |
| Insular Cortex_L | 3.612 | 0.061 | 0.046 |
| Insular Cortex_R | 11.921 | 0.001 | 0.137 |
| Cingulate_L | 11.981 | 0.001 | 0.138 |
| Cingulate_R | 6.108 | 0.016 | 0.075 |
| Hippocampus_L | 7.883 | 0.006 | 0.095 |
| Hippocampus_R | 8.828 | 0.004 | 0.105 |
| Caudate_R | 4.346 | 0.040 | 0.055 |
| Putamen_L | 5.094 | 0.027 | 0.064 |
| Precentral WM_L | 5.387 | 0.023 | 0.067 |
| Postcentral WM_L | 2.752 | 0.101 | 0.035 |
| Frontal WM_L | 10.366 | 0.002 | 0.121 |
| Frontal WM_R | 10.451 | 0.002 | 0.122 |
| Parietal WM_L | 6.440 | 0.013 | 0.079 |
| Parietal WM_R | 5.324 | 0.024 | 0.066 |
| Temporal WM_L | 7.942 | 0.006 | 0.096 |
| Temporal WM_R | 14.441 | ＜0.001 | 0.161 |
| Occipital WM_L | 6.052 | 0.016 | 0.075 |
| Occipital WM_R | 18.538 | ＜0.001 | 0.198 |

Covariates included age, BMI, and years of education. RK, radial kurtosis; HC, healthy control; L, left; OSA, obstructive sleep apnea; R, right; WM, white matter.
